# Macrophages in Recurrent Glioblastoma as a Prognostic Factor in the Synergistic System of the Tumor Microenvironment

**DOI:** 10.3390/neurolint15020037

**Published:** 2023-04-23

**Authors:** Nicola Montemurro, Bhavya Pahwa, Anish Tayal, Anushruti Shukla, Manuel De Jesus Encarnacion, Issael Ramirez, Renat Nurmukhametov, Vishal Chavda, Antonella De Carlo

**Affiliations:** 1 Department of Neurosurgery, Azienda Ospedaliero Universitaria Pisana (AOUP), University of Pisa, 56100 Pisa, Italy; 2 University College of Medical Sciences and GTB Hospital, New Delhi 110095, India; 3 Department of Neurosurgery, Russian People’s Friendship University, 121359 Moscow, Russia; 4 Royal Melbourne Hospital, Melbourne, VIC 3000, Australia; 5 Department of Spinal Surgery, Central Clinical Hospital of the Russian Academy of Sciences, 121359 Moscow, Russia; 6 Department of Pathology, Stanford of School of Medicine, Stanford University Medical Centre, Palo Alto, CA 94305, USA

**Keywords:** macrophages, glioblastoma, tumor microenvironment, prognostic factor, synergistic system, microglia

## Abstract

Glioblastoma (GBM) is a common and highly malignant primary tumor of the central nervous system in adults. Ever more recent papers are focusing on understanding the role of the tumor microenvironment (TME) in affecting tumorigenesis and the subsequent prognosis. We assessed the impact of macrophages in the TME on the prognosis in patients with recurrent GBM. A PubMed, MEDLINE and Scopus review was conducted to identify all studies dealing with macrophages in the GBM microenvironment from January 2016 to December 2022. Glioma-associated macrophages (GAMs) act critically in enhancing tumor progression and can alter drug resistance, promoting resistance to radiotherapy and establishing an immunosuppressive environment. M1 macrophages are characterized by increased secretion of proinflammatory cytokines, such as IL-1ß, tumor necrosis factor (TNF), IL-27, matrix metalloproteinase (MMPs), CCL2, and VEGF (vascular endothelial growth factor), IGF1, that can lead to the destruction of the tissue. In contrast, M2 is supposed to participate in immunosuppression and tumor progression, which is formed after being exposed to the macrophage M-CSF, IL-10, IL-35 and the transforming growth factor-ß (TGF-β). Because there is currently no standard of care in recurrent GBM, novel identified targeted therapies based on the complex signaling and interactions between the glioma stem cells (GSCs) and the TME, especially resident microglia and bone-marrow-derived macrophages, may be helpful in improving the overall survival of these patients in the near future.

## 1. Introduction

Glioblastoma (GBM) (World Health Organization Grade IV glioma) is a common and highly malignant primary tumor of the central nervous system in adults [1]. It accounts for approximately 14–15% of all brain tumors, with approximately 3–4 per 100,000 cases diagnosed worldwide annually [2]. The prognosis is extremely poor with a short overall survival (OS) of 12 months and a 5-year survival rate of less than 5% [3]. In cases of recurrence, a progression-free survival (PFS) of 10 months is usually expected, with an OS of about 22 months even after a second surgery [4,5]. The Cancer Genome Atlas (TCGA) network has classified GBM into four distinct molecular subtypes: classical, mesenchymal, pro-neural, and neural, based on a comprehensive assessment of genetic alterations and expression [6]. The current standard of care involves maximally safe resection of the tumor followed by radiotherapy and chemotherapy using temozolomide (TMZ). This is the Stupp protocol, which has been in use since 2005 [7]. Other novel therapies with promising effects on survival rates include monoclonal antibodies, innate immunotherapy, oncolytic viruses and small molecule inhibitors [8]. Immunotherapy, such as immune checkpoint blockade, chimeric antigen receptor T (CAR T) cell therapy, oncolytic virotherapy, and vaccine therapy, have provided fresh hope of improving the prognosis for GBM; ongoing studies are utilizing combinatorial therapies with the goal of reducing negative side effects and enhancing antitumor immune responses [9]. However, due to the unique intracranial environment of GBM, patients with recurrent GBM seem not to benefit from these novel therapies.

More and more recent papers are focusing on understanding the role of the tumor microenvironment (TME) in affecting tumorigenesis and subsequent prognosis [9,10,11,12,13], confirming the potential role of systemic inflammation indexes in patients with GBM [14]. The TME shown in patients with GBM is unique and, even if it is usually genetically stable, some changes in genetic profiles can occur [4,15]. Both non-immune and immune cells contribute to the highly immunosuppressive ”cold” TME phenotype [16]. Suppressive and pro-tumorigenic myeloid cells that represent a vast majority of myeloid cells in GBM TME actively contribute to the resistance of GBM to immunotherapy [17]. However, there still are largely unanswered questions regarding how GBM governs the metabolic and epigenetic landscapes of myeloid cells, as well as the mechanisms of the dynamic heterogeneity of these cells during immune and therapeutic responses in the context of GBM.

The components of TME include myeloid cells such as glioma-associated macrophages (GAMs), bone-marrow-derived macrophages (BMDMs), myeloid-derived suppressor cells (MDSCs), tumor-associated neutrophils (TANs), but also resident microglia, dendritic cells, lymphoid cells including CD8+ cytotoxic T cells, T-helper cells, T-regulatory cells and non-immune cells such as neurons, astrocytes, pericytes and endothelial cells [18,19]. The myeloid derivatives are responsible for the release of inflammatory cytokines and chemokines, whereas lymphocytes contribute to tumor cell lysis [20]. Changes are also observed in the extracellular matrix, blood–brain barrier, central nervous system (CNS) resident cells, GBM cells and glioma stem cells (GSCs) [21]. In the early stages, innate and adaptive immune responses are responsible for maintaining the host defense against the growing cell mass, but in the later stages there is a shift towards immune evasion and thus the facilitation of tumor development [22], acting like a double-edged sword, as Wu et al. [23] correctly defined it.

GAMs are the most numerous non-neoplastic cells in the GBM TME, constituting about 40% of the GBM tumor [24,25]. They originate either in the brain or are bone-marrow-derived macrophages [26]. Typically, they show differential activation as M1 (pro-inflammatory) or M2 (suppressive) macrophages, but this distinction is not enough to account for their highly variable behavior and molecular heterogeneity [17]. The presence of macrophages in the TME of recurrent GBM is directly related with the grade of tumor, as a higher infiltration of macrophages is often seen in GBM cases with poorer prognosis and worse overall survival (OS) [25,27,28,29]. Multiple signaling molecules, growth factors, transcriptions factors and epigenetic and post-transcriptional modifications affect the phenotype and activation state of GAMs. Unpolarized macrophages (M0 state) can be activated and polarized into the proinflammatory M1 and anti-inflammatory M2 subtypes in response to tumoral activation. In the TME, monocytes assume activation states that result from an adaptation to both the CNS and the TME [29].

In this review, we assess the impact of macrophages in the TME on the prognosis in patients with recurrent GBM. To simplify the process of understanding, we divided this review into three parts: pathology and molecular mechanisms of GBM, laboratory (which mainly involves pre-clinical and in vitro studies), clinical and therapeutic evidence of the current literature of the past six years.

## 2. Methods

**Figure 1:**
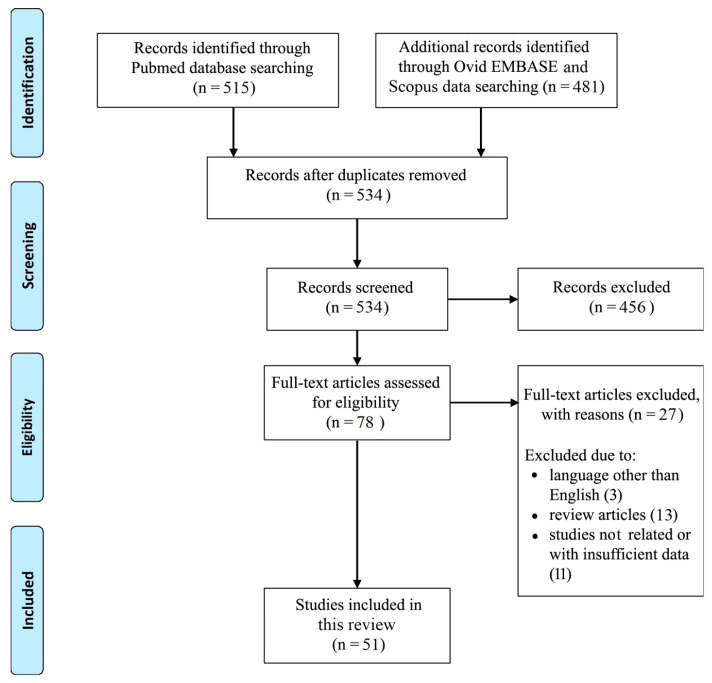
PRISMA flow diagram.

A PubMed, Ovid EMBASE and Scopus review was conducted to identify all studies dealing with macrophages in the recurrent GBM microenvironment. The following search terms were used from January 2016 to December 2022: macrophages, GBM, tumor microenvironment, prognostic factor, clinical and therapeutic evidence. A total of 534 articles, including those listed in the references of the retrieved studies, were found originally. We then excluded the following items: all publications not dealing with macrophages in the recurrent GBM microenvironment; all studies differing from the original articles (e.g., case reports/case series, letters, commentaries); non-English-written papers; and any other publication that did not comply with the goal of the present review. Further relevant references were identified from the bibliography of extracted articles as needed. After this process, a total of 51 studies was included in this review. Figure 1 shows all the details.

## 3. Macrophages in GBM Development and Pathophysiological Mechanisms

New research revealed that macrophages, which include bone-marrow-derived macrophages and brain-resident microglia, also known as GAMs and GAM-like cells, play a crucial role in tumorigenesis, changing treatment resistance, and creating an immunosuppressive environment [22,30]. Microglia ensure the functional equilibrium of the intracranial environment in physiological conditions by monitoring environmental changes, immunological surveillance, and homeostatic regulation [31]. Macrophages share some similarities and differences with microglia. In response to a pathological stimulus, the circulating monocytes in blood migrate to the CNS and differentiate into tissue macrophages, whereas microglia arise from progenitors of the yolk sac, during early embryonic development. Although they have different origins, they are hard to distinguish owing to a similarity in biomarkers such as IBA-1, CD 11b, CD 45, F4/80, CD 68, and CX3CR1. Differences are mentioned in Table 1.

**Figure 2:**
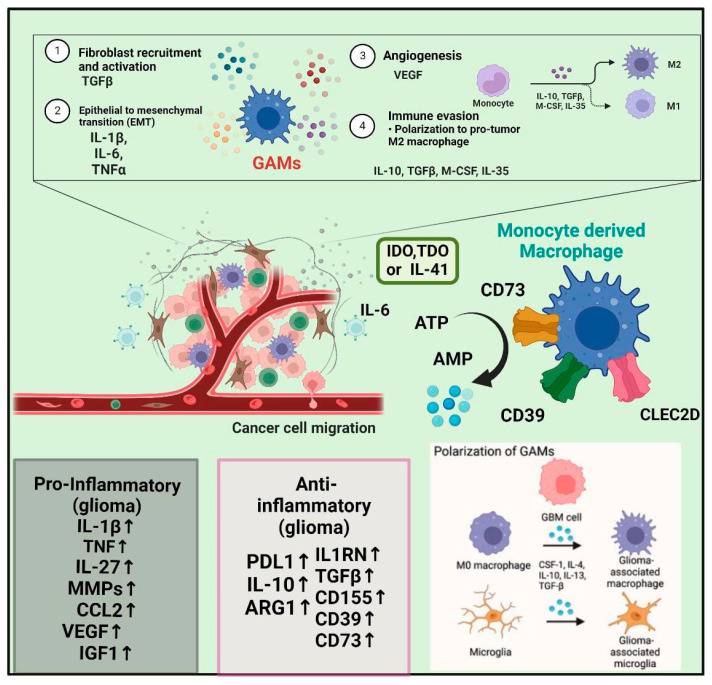
This figure shows how glioblastoma is associated with a highly immunosuppressive tumor microenvironment. (TME). Glioma-associated macrophages and microglia (GAMs) are a dominant population of immune cells in the GBM TME, contributing to the majority of GBM symptoms, including immunosuppression. GAMs in GBM include brain-resident microglia and macrophages, which include CD73, CD39, CLEC2D, and more. M1 (Anti-tumor) cells to M2 cells (GAMs) transition is also known as the pro- and anti-inflammatory glioma which represents the GBM progression and activated biomarkers shown in the above image.

The presence of GAMs accounts for 30% in the TME and favors the growth of the tumor [42,43,44,45]. Endothelial cells and GAMs release pro-inflammatory interleukin (IL)-6, which has been implicated in several pro-tumoral processes in GBM; as it enhances the activity of phosphoglycerate kinase 1 (PGK1) by promoting its phosphorylation, it reinforces GBM metabolic dependence on aerobic glycolysis and promotes macrophage recruitment by upregulating CCL5/CXCL5 and supports their alternative activation via PPAR/HIF-2 signaling [46] (Figure 2).

The malignant phenotype of recurrent GBM is promoted by the several cytokines released by GAMs, including tumor malignancy, angiogenesis, and resistance to therapies such as TMZ chemotherapy, radiotherapy, anti-angiogenesis, and immunotherapy. GAM recruitment is enhanced as a result of the production of human interferon (IFN) by infiltrating GAMs, epigenetic immunoediting, and continued expression of the myeloid-associated genes in GBM cells [22]. Loco-regional metabolic signals produced in tumor environments (glucose, glutamine, cysteine, lactate, Indoleamine-2,3-dioxygenase (IDO), adenosine, itaconic acid, acidic pH) may also influence the immunosuppressive functions of GAMs [47]. Indoleamine-2,3-dioxygenase (IDO) as well as tryptophan-2,3-dioxygenase (TDO) are important in tumoral cells defense against immunity response. The upregulation of IDO is in recurrent and primary GBMs associated with a decreased overall survival [47]. Nevertheless, GBM cells modify microglia phagocytic activity by changing several signaling pathways and epigenetic processes via the production of distinct surface and secreted chemicals. Therefore, a prospective anticancer treatment against GBM involves modulating and reeducating the population of microglia.

## 4. Laboratory Evidence and the Microglia/Macrophages TME

Macrophage migration inhibitory factor (MIF) is now emerging as a prospective anti-angiogenic therapy in GBM as high levels of MIF have recently been linked to tumor recurrence and poor survival [48,49,50]. It has been demonstrated that the GBM TME, which promotes vasculogenic structure, releases hypoxia-induced MIF, and that its expression is correlated with that of vascular endothelial growth factor (VEGF) [51]. In the recurrent GBM microenvironment, GAMs represent the major population, with up to half of the cells in the tumor mass [52]. The complex crosstalk between GSC and GAMs recruits more GAMs to the TME. Furthermore, GSCs polarize the recruited macrophages towards the M2 phenotype, the pro-tumoral subtype of GAMs, to make them their battle companion. De Groot et al. [53] reported that recurrent GBMs were shown to have few T cells and few immunological activation markers, while the TME is noticeably enriched for CD68+ macrophages. A subpopulation of cells has been discovered with a strong, opposing link with programmed cell death-1 (PD-1) signaling, which may correlate with their response to PD-1 inhibition [52], whereas core GAMs are evolving towards a pro-inflammatory state. A lack of T cells within the TME and a predominance of CD68+ macrophages prevent pembrolizumab anti-PD-1 monotherapy from inducing an effective immunologic response in the majority of GBM patients [52].

Liu et al. [54] reported that polyploid giant cancer cells and their daughter cells enhanced the polarization of GAMs into the M2 phenotype, with relevance to immunosuppression and malignancy in GBM. This can be partially attributed to the miR-340-5p-macrophage feedback loop [55]. Downregulation of miR-340-5p targets POSTN results in the recruitment of GAMs through integrin αvβ3. In addition, it promotes polarization of GAMs to the M2 macrophage phenotype by directly targeting LTBP-1. These activated GAMs secrete the transforming growth factor-β (TGF-β) which leads to HMGA-2 expression and inhibits miR-340-5p expression in GBM cells. Patients with low-miR-340-5p expression (*p* < 0.0001), high CD163 (*p* = 0.0002), high POSTN (*p* = 0.0003), high LTBP1 levels (*p* = 0.0006), and high HMGA-2 (*p* = 0.0157) have poor outcomes with shorter OS. Leucine-rich repeat-containing membrane protein-15 (LRRC15) is a transmembrane protein which is highly expressed in cancer cells of mesenchymal origin, and it is known to play a role in tumor invasion, metastasis and immunomodulation of TME [56]. In recurrent GBM samples, LRRC15 expression is significantly associated with macrophage markers, including integrin subunit αM (IT-GAM/CD11b) (*p* < 0.001), allograft inflammatory factor 1 (AIF1) (*p* < 0.05) and CD68 (*p* < 0.001) [57]. Subgroup analysis revealed a positive correlation of LRRC15 expression with the marker of M2-like macrophages, that is, CD206 (*p* < 0.001), CD163 (*p* < 0.001) and CD204 (*p* < 0.001). However, no relation was established with M1-like macrophage markers. This is suggestive of the role played by the LRRC15 gene in the recurrence of GBM with the involvement of M2 GAMs. Similarly, Hudson et al. [58] established that there was an upregulation of the M2 subtype of GAMs in recurrent GBM tissue. M1 macrophages are characterized by increased secretions of proinflammatory cytokines, such as IL-1ß, tumor necrosis factor (TNF), IL-27, matrix metalloproteinase (MMPs), CCL2, VEGF, and IGF1, that lead to the destruction of the tissue.

In contrast, M2 is supposed to participate in immunosuppression and tumor promotion, which are formed after being exposed to macrophage M-CSF, IL-10, IL-35 and TGF-β). The TME is determined by the overall number of GAMs in GBMs. By producing inhibitory cytokines and chemokines to the antitumor immune response, the M2-like GAM subtype supports the development of an immunosuppressive response. M2 GAMs support tumor progression by supporting tumor growth, invasion, immune evasion and promoting resistance to radiotherapy [59]. Otani et al. [60] studied the role of neurogenic locus notch homolog protein 1 (NOTCH 1) in signaling an immune evasion with post-oHSV (oncovirus-herpes simplex virus) viral therapy in patients with GBM. Their mode of action included RNA sequencing, TCGA analysis, flow cytometry, luminex- and enzyme-linked immunosorbent assay (ELISA)-based assays, animal models, and serum analysis of recurrent GBM patients. o-HSV infection significantly induces Jag-1 (NOTCH ligand) expression in infiltrating myeloid cells (*p* < 0.001), which leads to a cascade of events involving NOTCH activation in TME and the subsequent secretion of CCL2 by the macrophages, augmenting MDSCs. This causes a decline in anti-tumor immunity and, therefore, increases the chances of recurrence in a short time. The increase in serum CCL2 and IL-10 titers in these patients further consolidates the above observation. Retinaldehyde dehydrogenase-2 (ALDH1A2) is highly expressed in M2 GAMs in patients with recurrent GBM, whose expression increases with tumor recurrence at the gene and protein level [6]. ALDH1A2 is enzymatically broken down into retinoic acid (RA), that amplifies the production of MMP 2 and 9 in macrophages, facilitating the progression to more invasive phenotypes of GBM.

## 5. Therapeutic Evidence and Experimental Models

The standard of care for GBM is gross total surgical resection followed by a combination of radiotherapy and chemotherapy, whereas a second surgery and a re-irradiation can improve the OS [4,61,62,63,64]. However, GBM virtually always recurs with a poorer prognosis after first-line medical management; recent literature advocates the role of GAM in treatment resistance and recurrence. Pleiotrophin (PTN) is implicated in the self-renewal of recurrent GBM stem cells after surgical resection of the tumor. High PTN expression is associated with shorter OS and has been identified as an independent prognostic factor in patients with GBM. Recurrent tumors express elevated levels of PTN and are secreted by both tumor cells and GAMs [65,66,67,68]. PTN may promote tumor cell proliferation, self-renewal, and stem cell programming, whereas GAMs secrete the proangiogenic molecules CXCL2, which may contribute to angiogenesis, tumor growth and TMZ resistance [25,69,70,71,72,73]. These results show that the synergism of anti-angiogenic therapy and TMZ can be capitalized on to achieve favorable patient outcomes. The combi-therapy with TMZ and CXCR2-antagonization has an insignificant effect on the tumor volume [74]. However, the ratio of apoptosis to proliferation was significantly higher in the cohort with combination therapy in comparison to those with either TMZ alone or control [74]. Therefore, the combination of TMZ with CXCR2-antagonization represents a new promising treatment approach to overcome CXCR2-mediated resistance. Anti-programmed cell death 1 (PD1) antibody, pembrolizumab was unsuccessful in eliciting an effective immunological response in recurrent GBM patients attributed to the preponderance of CD68 macrophages [53]. IPI-549 (eganelisib) is a selective PI3K-gamma inhibitor and it was found to inhibit M2 macrophages, which suggests a negative correlation between M2 macrophages and OS [75]. A combination therapy of anti-PD ligand 1 antibody and IPI-549 increased the OS to 60% versus 0% in murine models who were administered the anti-PDL1 antibody alone. Similarly, other therapies targeting CD68+ macrophages, such as CSF1 inhibitors, were trialed but they did not produce therapeutic results, which is not unexpected given that macrophage cell modulation would need to be combined with strategies that induce T-cell activation, trafficking and effector activity within the tumor. Macrophage-derived insulin-like growth factor 1 (IGF-1) has been observed to contribute to the resistance of GBM to CSF-1R inhibitors, leading to a recurrence and decreased OS rates. Inhibiting this receptor restores the sensitivity of GBM to CSF-1R inhibition in recurrent tumors, prolonging the OS significantly [76].

Otani et al. [60] found that blockage of the NOTCH signaling pathway coupled with o-HSV viral therapy led to a reduction in the release of the cytokines secreted by macrophages in the TME, as well as recruitment of MDSCs. GBM with diffusely expressed CD204+ GAMs is usually associated with MGMT-promoter methylation [77]. Although this association is poorly understood, CD204+ GAMs may neutralize the effect of the MGMT-DNA protein to lose its function, which contributes to tumor progression. This relationship has no significant impact on the patient’s PFS after different treatment modalities. Liu et al. [78] demonstrated the role of insulin-like growth factor binding protein 2 (IGFBP2) in promoting the mesenchymal phenotype of recurrent GBM. When this protein was inhibited, there was a decline in the proportion of CD163+ M2 macrophages. Furthermore, there was significant suppression of tumor growth as well as improved OS.

A comparative study in orthotopic allograft mouse GBM models was conducted wherein the control group received fractionated external beam radiotherapy (XRT) and the other received a dual therapy comprising XRT and UNC2025 (XRT/UNC2025), which is a MerTK receptor inhibitor [79]. Although median survival was similar in both groups, bioluminescence imaging (BLI) showed significant growth delay with XRT/UNC2025 treatment and complete responses in 19%. In contrast, only 2% of 98 GBM mice of the same model treated with XRT survived 50 days and none survived 60 days [79].

Radiotherapy after surgical resection is an important and cardinal point of standard of care for GBM; however, Yoo et al. [80] showed the role of radiotherapy in switching the GBM phenotype to the mesenchymal type, which is associated with high recurrence and poor survival rates. A convincing explanation lies in the production of soluble intercellular adhesion molecule-1 (sICAM-1) and CSF-1 which increases the infiltration of macrophages as well as stimulating them to secrete a wingless-type MMTV integration site family, member 3A (WM3A) which causes a mesenchymal shift. Hence, radiotherapy must be combined with sICAM-1 and/or CSF-1 inhibitors.

During tumor developing, GAMs exert their protumor genic functions through various cytokines, including TGF-β, IL-6, IL-10. These immune cytokines promote the invasion of GBM cells. In hypoxic areas, GAMs promote angiogenesis with the secretion of multiple angiogenic factors that induce the M2 polarization of GAMs. Hypoxia induces the expression of hypoxia-inducible factor 1-alfa (HIF-1-alfa) in GAMs, which upregulates VEGF and VEGFR. VEGFR on endothelial cells stimulate the secretion of MMP that destroys the basement membrane and extracellular matrix components; this in turn disturbs the endothelial–pericyte contact and promotes the migration and the proliferation of endothelial cells [17,22]. VEGF prevents dendritic cell maturation, restricts T-cell recruitment into tumors, or promotes T-cell exhaustion. TGF-β production in GAMs and M2-like polarization are stimulated by VEGF/VEGFR signaling. In addition to VEGF, angiopoietin-2 (ANGPT2) impairs tumor defense by inducing Tie-2-expressing mono-cytes/macrophages to release IL-10, which promotes the growth of T-regulatory cells [22].

Tumor angiogenesis is a hallmark of neoplasm and is essential for providing nutrients and oxygen to the malignant tissue. GAMs serve as a major source of angiogenic factors, such as VEGF, boosting the angiogenic switch. However, clinical trials have shown that anti-VEGF/anti-vascular endothelial growth factor receptor-2 (VEGFR2) treatment has little impact on OS [81,82,83,84,85]. Rather, in patients with recurrent GBM treated with anti-angiogenic therapy, an increase in CD11b+ cells was observed, which correlates inversely with OS. Min et al. [86] reported that anti-VEGFR2 therapy may have therapeutic potential to control the immune inhibitory functions of mesenchymal-associated tumor-associated macrophages (MA-TAMs) in colorectal cancer, resulting in enhanced efficacy of immunotherapy with immune checkpoint inhibitors. Therefore, anti-VEGF/VEGFR therapy should not be used as a standalone therapy, especially in patients with recurrent GBM [28,87,88,89]. Invariant natural killer T (iNKT) cells, which recognize glycolipid ligands presented in CD1d molecules and respond to a synthetic glycolipid, play an important role in anti-tumor immunity [90,91]. At present, GBM has not been recognized as a target for immunotherapy with iNKT cells [90], but CD1d expression by several glioblastoma cell lines has been reported [90,92]. However, because iNKT cells exhibit direct cytotoxicity against CD1d-expressing GBM cells, some authors propose that the adoptive immunotherapy of iNKT cells should be an ideal therapeutic strategy for GBM [90,93]. In addition to its immediate natural cytotoxicity, which is activated by the stimulation of germline-encoded cell surface receptors, iNKT cells contribute to cancer-immune surveillance modulating T-cell-mediated antitumor immune responses by preserving the quality of dendritic cells and enhancing the presentation of tumor antigens [93]. In a first-in-human phase I trial performed at our institution, 10 patients with recurrent GBM were treated with a single dose of peripherally infused EGFRvIII-directed CAR T cells [94], and, while the exploration of CAR T-cell therapy in GBM has just begun, early results have demonstrated the feasibility, safety, and even signs of efficacy of using this approach.

## 6. Clinical Evidence and Leukocyte Infiltrates in Recurrent GBM

Regarding GBM tumor subtypes, macrophage and microglia signatures have been reported to be highly enriched in GBM patients with mesenchymal and neural subtypes, respectively [61]. Subtypes of recurrent or primary GBM with a higher percentage of macrophages are associated with worse patient outcomes. Liu et al. [44] demonstrated that the magnitude of M2 GAM infiltration is significantly correlated with poor OS in recurrent GBM patients. Conversely, Wang et al. [62] found that high levels of GAMs in patients with newly diagnosed GBM did not correlate with OS. The pro-tumoral macrophages interact with a multitude of cells in the TME to endorse tumor progression. For instance, GAMs interact with CD8+ T cells to induce an anergic state, thus, leading to a negative correlation between the percentage of CD8+ T cells in TME and patient prognosis. In addition, GAMs modulate the surrounding TME with the help of macrophage receptors with a collagenous structure (MARCO) to induce a phenotypic shift towards the mesenchymal subtype, promoting both invasive and proliferative activities, as well as radiotherapy resistance [63,64]. MARCO^high^ GAMs also promote tumor engraftment and growth in vivo. Moreover, both MA-TAM master regulators and their target genes, including MARCO^high^, are significantly correlated with reduced OS (*p* < 0.0001). Both GAMs and MDSCs have the ability to attract regulatory T lymphocytes into the tumor, but MDSCs prevent the activation of both cytotoxic CD4+ T helper cells and CD8+ T cells, as well as the NKT cell-mediated cytotoxic responses [27]. The presence of regulatory T cells may further contribute to a lack of effective immune activation against recurrent GBM. Magri et al. [65] took peripheral blood samples and tumor tissue samples in patients with primary and recurrent GBM and observed that patients with recurrent GBM had a higher proportion of macrophages and infiltrating lymphocytes, together with exhaustion markers. Elsewhere, Fu et al. [66] showed that the percentage of GAM observed in recurrent GBM samples was lower than that in primary GBM samples. Table 2 shows more recent therapeutic strategies for the management of GAMS in GBM patients.

## 7. Conclusions

GAMs make up a very important amount of the recurrent GBM microenvironment. Their presence is reflective of tumor aggressiveness and, consequently, of the OS of those patients with GBM, as differential polarization of macrophages into M1 and M2 states affects prognosis and outcomes. In particular, the numbers of macrophages are increased in the tumor, and upon recurrence, there is a higher macrophage-to-microglia ratio [95,96]. Pro-tumoral M2 polarization correlates with a worse prognosis and a short OS in patients with GBM, due to the development of immunosuppressive and angiogenic states, which is mediated by a wide array of genes and their associated signaling pathways. In cases of recurrence, the total amount of these cells is relatively lower, but it is still significant. Specific phenotypes of recurrent GBM show invasive and proliferative properties as well as a resistance to radiotherapy due to their macrophage component. GAMs act critically in enhancing tumor progression and can alter drug resistance, promoting resistance to radiotherapy and establishing an immunosuppressive environment.

Because there is currently no standard of care in recurrent GBM, novel identified targeted therapies based on the complex signaling and interactions between the GSCs and the TME, especially resident microglia and BMDMs, may be helpful in improving the overall survival of these patients in the near future.

The recent advances in the understanding of mechanisms of myeloid cell-driven immunosuppression as well as of mechanisms of recruitment and localization of myeloid cells will be beneficial when we design new therapeutic approaches. Given a growing and ever more interested understanding, more specific genetic and molecular alterations will pave the way for improved therapeutic management and better outcomes in the current diagnosis and poor management of GBM.

## Figures and Tables

**Table 1:** Variables of the present study.

Parameter	Macrophages	Microglia
Origin [32,33,34]	Monocytes	Yolk Sac
No. of ramified processes	Fewer	More
Location in tumor	Tumor core	Tumor periphery
Rate of infiltration in tumor [35]	Slow	Rapid
Expression of various markers		
CD45 [36]	High	Low
CCR2 [37]	High	Low
CD 49 [37]	High	Low
CX3C-Receptor 1 [38]	Low	High
Green Fluorescent Protein [38]	Low	High
R26^Reporter^ [39,40]	Temporary	Permanent
Pro-inflammatory cytokines(II1a and II1b) [41]	High	Low

**Table 2:** Therapeutic strategies for the management of GBM that act by inhibiting GAMs.

S. No.	Author; Year	Drug	Mechanisms
1.	Quail et al., 2016 [36]	CSF-1 inhibitors	CSF-1 promote macrophage survival and differentiation
2.	Quail et al., 2016 [36]	IGF-1 inhibitor	Restore the sensitivity of GBM to CSF-1R in hibition inrecurrent tumors
3.	Otani et al., 2022 [23]	NOTCH signaling pathway inhibitor	Reduction in the release of the cytokines secreted by GAMs and recruitment of myeloid-derived suppressor cells
4.	Liu et al., 2019 [38]	IGFBP2 inhibitors	Promote mesenchymal phenotype of GBM and decline in the proportion of CD163+ M2 macrophages
Combination therapy			
1.	Urbantat et al., 2021 [33]	TMZ and CXCR2-antagonist	TMZ induces cell cycle arrest and CXCR2-antagonist inhibits angiogenesis
2.	Miyazaki et al., 2020 [35]	Anti-PD ligand 1 antibody and IPI-549	Anti PD ligand 1 antibody disinhibits immune system and IPI-549 inhibits M2 macrophages
3.	Quail et al., 2016 [36]	Radiotherapy and UNC2025	UNC2025 causes tyrosine kinase receptor inhibition

CSF-1: Colony stimulating factor-1; CSF-1R: Colony stimulating factor-1 receptor; IGF-1: Insulin-like growth factor-1; IGFBP2: Insulin-like growth factor binding protein 2; NOTCH: Neurogenic locus notch homolog protein 1; TMZ: temozolomide.

## Data Availability

Not applicable.
